# Multiple organs involved in the pathogenesis of non-alcoholic fatty liver disease

**DOI:** 10.1186/s13578-020-00507-y

**Published:** 2020-12-07

**Authors:** Xiaoyan Li, Hua Wang

**Affiliations:** 1 Department of Oncology, the First Affiliated Hospital of Anhui Medical University, Hefei, 230022 China; 2 Inflammation and Immune Mediated Diseases Laboratory of Anhui Province, Hefei, 230032 China

**Keywords:** Non-alcoholic fatty liver disease, Energy metabolism, Lipid, Hormone, Inter-organ crosstalk

## Abstract

Non-alcoholic fatty liver disease (NAFLD) represents the leading cause of chronic liver disease worldwide and the anticipated health burden is huge. There are limited therapeutic approaches for NAFLD now. It’s imperative to get a better understanding of the disease pathogenesis if new treatments are to be discovered. As the hepatic manifestation of metabolic syndrome, this disease involves complex interactions between different organs and regulatory pathways. It’s increasingly clear that brain, gut and adipose tissue all contribute to NAFLD pathogenesis and development, in view of their roles in energy homeostasis. In the present review, we try to summarize currently available data regarding NAFLD pathogenesis and to lay a particular emphasis on the inter-organ crosstalk evidence.

## Introduction

Non-alcoholic fatty liver disease (NAFLD), has been commonly considered as the leading cause of chronic liver diseases in Western countries over the last decade [1]. In the meantime, urbanization in many developing countries has recently led to a sedentary lifestyle and overnutrition, which contribute to obesity, metabolic syndrome and the emerging condition of NAFLD [2]. NAFLD currently has a reported prevalence of 12–38% worldwide and the number is growing inexorably and steadily along with the unprecedented levels of obesity in human society [3]. On account of the radical cure of hepatitis C, the imminent disappearance of hepatitis B and the uncontrolled energy-dense lifestyle, NAFLD would undoubtedly become the mainspring for liver related morbidity and mortality very soon. As estimated, NAFLD should be the most frequent indication for liver transplantation by 2030 [4]. To date, no drug for NAFLD has received FDA approval, giving rise to insufficient pharmacotherapeutic interventions in clinical practice [5]. These alarming situations necessitate the extension of our understanding towards pathogenic mechanisms of NAFLD.

NAFLD could be simply interpreted as the condition where excess fat is stored in the liver, and this secondary fat accumulation is not the consequence of other factors like heavy alcohol consumption, drug side effects or genetic variations [6]. Whether liver inflammation exists is the basis to subdivide NAFLD into two types, fatty liver and non-alcoholic steatohepatitis (NASH), and the latter is the progressive phenotype of NAFLD spectrum [7]. Persistent inflammation will jeopardize liver homeostasis and activate the repair processes. Activated hepatic stellate cells (HSCs) secrete extracellular matrix (ECM), including type 1 collagen, to support the injured region and structure the regeneration scaffold for subsequent hepatocyte proliferation (i.e., liver fibrosis formation), along with the clearance of necrotic tissues by infiltrated leukocytes [8]. This ‘wound-healing’ process will end with the settlement of activated HSCs, ECM resolution and revascularization, given the detrimental effects are only transient [9]. Hence, it’s objective to regard liver fibrosis as a well-orchestrated advantageous response towards hepatic injury, while the ongoing insults against liver should be to blame for disease progression to cirrhosis or hepatocellular carcinoma (HCC) [10]. Specifically, for NAFLD, the persistence of liver steatosis derives from the overload of free fatty acids (FFAs) influx, which is the coefficient result of high fat diet, obesity, IR, gut microbiota alteration, and other potential risk factors. Furthermore, high levels of FFAs will exert the lipotoxic effects, causing endoplasmic reticulum (ER) stress and mitochondrial dysfunction (oxidative stress, production of reactive oxygen species (ROS), etc.) in the liver [11]. IR not only motivates hepatic de novo lipogenesis (DNL), but also causes adipose tissue dysfunction with consequent adipokines and inflammatory cytokines production [12]. Gut microbiota dysregulation could lead to the increase of intestine permeability and the release of proinflammatory cytokines into circulation [13]. Multiple pathways synergistically create a vicious cycle which slowly exacerbates the disbalance of liver homeostasis and induces the shift from simple steatosis to chronic inflammatory state of NASH. Based on evidences above, NAFLD is definitely not a single-organ disease, but more like the hepatic manifestation of a variety of complicated metabolic disorders involving different organs and systems. Here we will review recent findings about brain, gut, adipose tissue and liver interactions in NAFLD pathogenesis and try to get a relatively comprehensive understanding of the disease mechanism.

### Hepatic pathogenesis of NAFLD

The initiating events in NAFLD arise from the development of obesity and IR at the level of the adipose tissue and liver. The dysregulation of peripheral lipolysis, DNL, and dietary fat cause the increased FFA flux within the liver and then place hepatocytes under the lipotoxic condition. These lipotoxic FFAs are partitioned into inert intracellular triacylglycerol (TAG) for storage via acyl-CoA synthetic activity and mitochondrial β-oxidation, and the accumulation of TAG in hepatocyte cytoplasm, which is also called steatosis, is the generally accepted histological hallmark of NAFLD [14]. Many patients could stay in the NAFL stage for years, while the chronic insults would ultimately exceed the hepatic capacity to deal with the overload of fatty acids (FA) [5]. Significant hepatocyte injury leads to cell injury and inflammation, subsequently bringing Kupffer cells and other immune cells to the battlefield. Uncontrollable lipotoxicity facilitates ROS formation, ER stress, and hepatocellular dysfunction. Immune and apoptotic pathway activation results in cell death, which further drives fibrosis development over time [15].

Two important enzyme systems, acetyl-CoA carboxylase (ACC) and fatty acid synthase (FAS), play vital roles in Hepatic FA synthesis. Insulin and glucose could regulate both enzymes by activating sterol regulatory element-binding protein 1c (SREBP-1c) and carbohydrate-responsive element-binding protein (ChREBP), two important transcriptional factors in DNL, respectively. Liver X receptor (LXR), a nuclear receptor, also directly controls the activity of both SREBP-1c and ChREBP by binding to response elements in the promoters of genes [16]. Deletion of LXR leads to decreased SREBP-1c expression and less lipogenesis in mice. The induction effects of LXR on ACC and FAS could also be indirectly influenced by insulin and glucose [17]. As hyperinsulinemia and hyperglycemia is commonly seen in NAFLD population, the link between glucose and lipid metabolism is conspicuous. Excess glucose is usually stored as glycogen, mediated by insulin, while it could also be esterified into TAGs via DNL. Hepatic IR in NAFLD is found mediated by proinflammatory cytokines, ER stress, apoptosis pathways, and even lipid metabolites. Accumulation of diacylglycerol (DAG), a lipotoxic intermediate of FA synthesis, within cytosolic lipid droplets induces translocation of protein kinase C (PKC)-ε to the plasma membrane and inhibits the intracellular kinase domain of the insulin receptor. In a NAFLD cohort, DAG content and PKCε activation are the most significant predictors for hepatic IR and associate with 60% of the variability in hepatic insulin sensitivity [18]. Glucocorticoid receptor (GR) of hepatocyte is found critical for both direct and indirect transcriptional regulations of IR, hyperglycemia, gluconeogenesis and fatty liver [19]. A recent study targeting 17-hydroxyprogesterone (17-OHP), an intermediate product of steroid synthesis, pointed out the involvement of hepatic Cyp17A1/17-OHP/GR pathway in the development of IR [20].

The FA β oxidation process includes three major steps, activation, transportation and oxidation. Fatty acids are activated by cytosolic and ER acyl-CoA-synthetase to acyl-CoA for β oxidation or TAG synthesis. In charge of transporting acyl-CoA to mitochondria, carnitine palmitoyl transferase 1 (CPT1) serves as the rate-limiting enzyme for FA β oxidation and it could be inhibited by an important DNL intermediate, malonyl-CoA. This process could be inhibited by insulin and activated by peroxisome proliferator-activated receptor (PPAR)-α. In mitochondria, fatty acids are oxidized to produce energy for vital activities, and incidentally, to provide fatty acids with shorter chains and acetyl-CoA for other metabolic processes. Long-chain fatty acids are re-esterified in hepatocytes to produce TAG and stored in lipid droplets (LDs) or coupled to apolipoproteins and further secreted as very low-density lipoprotein (VLDL) [21]. When the TAG incorporation into VLDL is blocked by microsomal TAG transport protein (*MTTP*) and apolipoprotein B (*APOB*) mutations in patients, TAG accumulates in the liver and consequently causes hepatic steatosis and NASH development [22]. Another mechanism to remove heatic fatty acids is TAG synthesis. In this process, stearoyl-CoA desaturase 1 (SCD1), diglyceride acyltransferase (DGAT), ACC, and FAS are major enzymes whose expressions and activities account for disturbed TAG, fatty acids accumulation and NAFLD development. In the methionine-choline deficient (MCD) diet NASH model, the deletion of *Scd1* gene impairs TAG synthesis and induces hepatocyte apoptosis [23]. It’s also reported that inhibition of DGAT also leads to lipotoxicity [24] and liver specific DGAT overexpression increases VLDL secretion [25].

ROS production in hepatocyte is an incidental consequence of FAs oxidation. FAs overloading leads to upregulation of minor pathways (such as peroxisomal oxidation, microsomal oxidation and ER ω-oxidation) and mitochondrial respiratory chain. Consequent increase of ROS production surpasses normal antioxidative mechanisms like superoxide dismutase or glutathione, leading to oxidative stress and the initiation of NASH. NAFLD patients have lower hepatic expressions of genes related to mitochondrial biogenesis, peroxisomal proliferator-activated receptor gamma coactivator 1α (PGC1α), nuclear respiratory factor 1 (NRF1), and mitochondrial transcription factor A (mtTFA), which paves the way for disease progression [26]. ROS induces lipid peroxidation, damages plasma/intracellular membranes, and causes cell necrosis. 4-hydroxynonenal (4-HNE) and malondialdehyde (MDA) produced by lipid peroxidation promote inflammation and influence the posttranslational modifications (PTMs) of multiple hepatic proteins [27]. Many studies have discussed the ameliorating effects of radical-scavenging antioxidants in hospitalized NASH patients [28], backward suggesting the critical role of oxidative stress in NAFLD.

Evidence suggests that ER stress is among the most important factors for NAFLD pathogenesis [11]. The ER, an intracellular organelle, is sensitive to lipotoxicity. Dysregulation of ER function is represented by disturbed unfolded protein response (UPR), an adaptively orchestrated arrest of protein synthesis, which can further perpetuate ER stress. Subsequent oxidative and inflammatory signaling pathways trigger apoptosis and autophagy via PERK-mediated C/EBP homologous protein (CHOP), inositol-requiring enzyme 1α (IRE1α)-mediated recruitment of tumor necrosis factor receptor-associated factor 2 (TRAF2) and signal-regulated kinase 1/c-Jun N-terminal kinase (JNK). IRE1 also splices the transcription factor X-box binding protein 1 (XBP1), which activates JNK and inhibitor of κB kinase (IKK)-NFκB signaling, to modulate inflammatory cascades and ROS production [29]. Liver biopsy samples from NAFLD patients show a specific association between disease severity, spliced XBP1 mRNA and JNK phosphorylation. Compared with healthy individuals, NAFLD patients have a variable degree of UPR activation [30].

Apoptosis signal‐regulating kinase 1 (ASK1) is a member of the mitogen‐activated protein kinase kinase kinase (MAP3Ks) family and could activate downstream JNK and p38 mitogen-activated protein kinase (MAPK) signaling cascades to regulate autophagy, apoptosis, and inflammation. ASK1 itself could be activated by oxidative stress, ER stress and inflammatory cytokines [31]. Hepatic ASK1 activation is a key process in the progression of NASH and a promising target for treatment of the condition [32]. By modulating downstream p38-JNK1 and JNK2 signaling, ASK1 aggravates metabolic dysregulation of lipid and glucose, and precipitates hepatic inflammation [33, 34]. In the murine NASH model, one selective ASK1 inhibitor improves not only metabolic parameters but also hepatic steatosis, inflammation, and fibrosis [35]. A recent publication reported that ablation of *p38* gene in the liver could increase simple steatosis but attenuate oxidative stress-induced injury and fibrosis during NAFLD [36]. The detrimental and protective roles of p38 in different disease stages remind us that NAFLD therapies targeting ASK1-p38 pathway have to proceed with caution.

Hepatokines have also received considerable attention from researchers, considering their role in NAFLD pathogenesis [37]. Fetuin-A has long been considered as a liver-derived regulator for metabolic balance and is reported correlated with NAFLD in humans [38]. Fetuin-A contributes to the activation of Toll-like receptor 4 by fatty acids, which induces inflammation and IR. Hepatic fetuin-A binds to peripheral insulin receptors to inhibit insulin signaling [39] and correlates with key enzymes for lipid and glucose metabolism, such as SREBP-1c, CPT1, and phosphoenolpyruvate carboxy kinase 1 (PEPCK1) [40]. Many publications show significantly higher serum fibroblast growth factor 21 (FGF21) in NAFLD patients compared to healthy controls [41, 42]. FGF21 directly regulates lipid metabolism and reduces hepatic lipid accumulation in an insulin-independent manner. Toxic lipid accumulation in the MCD diet NASH model induces early FGF21 expression [43]. Adenovirus-mediated knockdown or genetic deletion of FGF21 cause lipotoxic damage, hepatic steatosis and dyslipidemia [44, 45]. FGF21 is also reported to mediate energy homeostasis via regulation of sweet taste [46]. Another major hepatokine, angiopoietin-like 8 (ANGPTL8), correlates with hepatic lipid content independent of IR in NAFLD patients. ANGPTL8 is activated by ER stress or hyperlipidemia, and hence leads to the inhibition of lipoprotein lipase activity, and the activation of autophagic process [47].

Finally, autophagy has also been suggested participating in NAFLD. In murine models and patients of NAFLD, autophagy activity decreases [48–51]. Loss of autophagic modulation against cell death leads to hepatic steatosis and the shift from NAFLD to NASH. Overexpression of *autophagy-related 7* (*Atg7*) or *Atg14*, key autophagy mediators, eliminates steatosis in high fat diet (HFD)-fed mice, while specific deletion of *Atg7* gene in the liver alters TAG secretion and increases hepatic lipid contents. Hyperinsulinemia and IR suppress hepatic autophagy and key autophagy genes such as *Atg12*, and *Gamma-aminobutyric acid type A receptor associated protein like 1* (*Gabarapl1*) [52]. Conversely, autophagy induction also ameliorates ER stress and IR in NAFLD mice [53, 54]. Here exists a vicious circle, in which hepatic steatosis and lipotoxicity-induced IR synergistically impair autophagy which further exacerbates steatosis and insulin sensitivity. Through autophagy, damaged mitochondria containing excessive abnormal intracellular components could be removed to prevent ROS production and apoptosis [55]. Once this process gets dampened, e.g., by *Atg5* knockdown, ROS and energy imbalance activates upstream JNK/c-Jun signaling, which sensitizes hepatocytes to cell death [54].

### Adipose tissue and NAFLD

Adipose tissue was considered originally as an inert organ only for fat storage, while recent studies have revealed its crucial roles participating in both energy homeostasis and immune regulation [56]. Altered adipose tissue biology has been recognized as a key early event in the initiation of NAFLD. During obesity progression, adipocytes gradually become hypertrophied in association with macrophages infiltration and subsequent adipokines, cytokines and chemokines alteration [57]. The expansion of adipose tissue also causes hypoxia and subsequent adipocyte death, triggering low-grade chronic inflammation, accumulation of ECM, and eventually IR. Local IR leads to more lipolysis in the adipose tissue and excess release of FFAs into the circulation [58]. The origin of hepatic TAG in NAFLD have been analyzed and indicate that adipose tissue lipolysis, DNL and dietary intake contribute to 60%, 25% and 15% of total hepatic TAGs, respectively [59]. Enhancement of ECM component level may incur progressive fibrosis in the adipose tissue, thereby limiting the fat storage capacity of adipocytes and promoting ectopic fat uptake [60]. Proinflammatory cytokines and chemokines produced by infiltrated macrophages could exacerbate IR and recruit more immune cells to the inflamed adipocytes [61]. It was reported in NAFLD patients that liver necroinflammation and fibrosis increased significantly with visceral fat in a dose-dependent manner [62]. Meanwhile, the adipose tissue from NAFLD patients were found with an increased expression of genes that regulate inflammation, and the adipose tissue macrophages produced increased levels of inflammatory cytokines, compared with control [63]. Inflamed adipose tissue macrophages were showed to signal to bone marrow and to stimulate production of myeloid cells, resulting in an exacerbation of inflammation and associated obesity processes [64, 65]. In a recent study, transplantation of adipose tissue from obese mice rapidly induced hepatic neutrophil recruitment by upregulating CXCL chemokine family genes and secondary macrophage accumulation [66]. Several studies have reported associations between adipocyte death and the pathogenesis of NFALD [67, 68]. After globally deleting the *Bid* gene or specifically deleting the *Fas* gene of adipocytes in HFD-fed mice, adipocyte death was mitigated, and as a result, IR and fatty liver were ameliorated. Correspondingly, adipocyte apoptosis caused by adipocyte-specific deletion of the synaptosomal-associated protein 23 (*Snap23*) gene led to IR and hepatic steatosis [69, 70]. The induction of adipocyte death in HFD-fed mice caused marked upregulation of *Mcp1*, which is important for macrophage recruitment in both adipose tissue and liver, at a very early time point. This rapid elevation of *Mcp1* was found to be mainly contributed by adipocytes, other than macrophages. After adipocyte death and macrophage activation, the elevation of epinephrine (EPI) and norepinephrine (NE) and the subsequent activation of lipolysis were observed [61].

The adipose tissue also serves as an important endocrine organ participating in energy balance by sensing metabolic signals and secreting a number of adipokines such as leptin, adiponectin, and resistin [71, 72]. The ‘energy expenditure hormone’ leptin is mainly secreted by visceral adipocytes and it’s involved in a range of energy modulating activities including hunger, food energy utilization, physical exercise, thermogenesis, and fat mass regulation [73, 74]. Leptin downregulates the transcription of the preproinsulin gene and insulin excretion, and high levels of leptin were documented in obese individuals or during IR [75, 76]. Proinflammatory cytokines such as IL-1 and TNF-α could increase leptin levels and subsequently perpetuate the loop of chronic inflammation in obesity [77, 78]. Especially in the liver, leptin suppresses the lipogenic process by lowering the expression of SREBP-1 [79, 80]. It exerts a permissive role in promoting liver inflammation and fibrosis by enhancing the production of type 1 collagen, upregulating tissue inhibitor of metalloproteinase (TIMP)-1 expression and downregulating matrix metalloproteinase 1 (MMP1) expression [81]. Leptin could also upregulate the expression of transforming growth factor (TGF)-β in Kupffer cells and sinusoidal endothelial cells, which contributes to liver fibrogenesis [82]. Although leptin has been reported contributing to IR, steatosis development and fibrogenesis, there are still disputes on its association with NASH severity in different cohorts [83, 84]. Hence, more studies with careful designs and strict criteria are needed to elucidate the role of leptin in NAFLD.

Another major adipokine is adiponectin, which is an adipocyte-derived anti-inflammatory mediator eliciting AMP-activated protein kinase (AMPK) signaling. Adiponectin suppresses adipose TNF-α expression and induces anti-inflammatory gene expression in leukocytes [85]. Suppression of adiponectin secretion by selectively deleting conventional kinesin heavy chain in adipose tissue exacerbates HFD-induced obesity and its associated metabolic disorders [86]. In NASH patients, circulating adiponectin levels were remarked diminished, and the downregulation of hepatic adiponectin could be reserved by weight loss [87]. A meta-analysis showed that hypoadiponectinemia serves as a critical feature of NASH patients and the reduction of adiponectin correlates closed with disease progression [88]. In an animal model of deleting C-terminus Hsc70-Interacting protein (CHIP), oxidative stress, IR, and hepatic inflammation had been achieved only except for steatosis because of a compensatory upregulation of adiponectin. By activating the AMPK-forkhead box O (FOXO)-signaling axis, adiponectin could override oxidative stress and JNK signaling, resulting in the counteracting progression of hepatic steatosis [89].

Resistin is mainly produced by adipocytes in mice and ATMs in humans [90]. Treatment of resistin in mice induced IR by impairing glucose tolerance and insulin action, and also upregulated suppressor of cytokine signaling 3 (SOCS3) expression, which further inhibited insulin signaling [91]. Knockout of resistin decreased hepatic steatosis via downregulating genes related to in hepatic lipogenesis and VLDL export [49]. Resistin has a proinflammatory role by not only stimulating multiple inflammatory cytokines (TNF-α, IL-1β, IL-6 and IL-12) [92], but also activating the MAPK pathway and the coagulation cascade. [93] Moreover, resistin could induce the production of TGF-β and type I collagen [94]. It was also reported that resistin could upregulate expressions of several chemokines [95]. Data about other adipokines (visfatin, retinol-binding protein 4, chemerin, acylation-stimulating protein, adipsin, apelin, obestatin, omentin, vaspin nesfatin-1, neopterin, neuregulin-4, etc.) are inconclusive or limited. Further studies may elucidate their roles in NAFLD.

In mammals, the adipose-tissue pool consists of white adipose tissue (WAT) and brown adipose tissue (BAT). Compared to WAT, BAT plays a distinct role in maintaining energy homeostasis because of its abundance in mitochondria and capillaries. BAT generates heat by catabolizing glucose and fatty acid, hence it acts as a protector against obesity and diabetes because thermogenesis dissipates excess energy from high-calorie intake [96]. Decrease of BAT activities could cause metabolic disorders including IR and type 2 diabetes mellitus (T2DM) [97]. In the subcutaneous inguinal WAT, there is a group of brown fat-like adipocytes called browning of WAT, which also contribute to thermogenesis. Transgenic expression or deletion of PR domain containing 16 (PRDM16) in WAT, a transcription coregulator for brown adipocyte development, could bi-directionally attenuate diet-induced obesity (DIO) or lead to obesity [98]. Reported in a recent study, deletion of AMPK in adipocytes led to BAT mitochondrial dysfunction, and secondarily exacerbated hepatic steatosis, IR, and glucose intolerance [99]. Reduction of adipose AMPK commonly observed in NAFLD patients, hence this study offered mechanism for NAFLD pathogenesis and a potential therapeutic target [100].

### Gut and NAFLD

Gut, or the gastrointestinal tract, plays a critical role in human physiology in terms of digestion, absorption of nutrients and the excretion of waste. Its mucosal barrier protects the body from pathogens and extrinsic antigens, and gut also secrets hormones to communicate with other organs. Another important profile is that trillions of microorganisms inhabit in the gut and they are closely involved in human metabolism, immune regulation, and behavior modulation. Recently, gut and gut microbiome are linked with the pathogenesis of NAFLD by more and more studies, and ‘gut-liver axis’ or ‘gut-brain-liver’ axis have also become hot topics among scholars [13, 101, 102].

Gut could initiate complex hormonal responses to changes in the nutritional status by secreting peptides like ghrelin, cholecystokinin (CCK), glucagon-like peptide 1 (GLP-1), fibroblast growth factor 19 (FGF19), etc., and some of these gut signals have also been considered as important mediators in NAFLD development [103].

Ghrelin is a small peptide mainly synthesized by the stomach and released into the circulation in two isoforms, the acylated ghrelin (AG) and the des-acyl ghrelin [104]. Ghrelin could stimulate appetite and act with the growth hormone secretagogue receptor (GHS-R). Apart from its unique role in the orexigenic central circuit, ghrelin has recently received considerable attention in studies of metabolic diseases and liver diseases due to the regulatory functions on immunity and disease pathogenesis independent of its effects on food intake [105]. The association between elevated AG levels and hepatic steatosis was documented in NAFLD patients [106]. Both animal and human studies showed that ghrelin could directly promote lipogenesis and inhibit lipolysis in adipocytes. Administration of AG inactivated AMPK signaling, stimulated TAG storage in the liver and caused dysregulation of lipid oxidation and mitochondrial function [107]. By deleting ghrelin or ghrelin receptor genes in mice, the facilitation on de novo lipogenesis was eliminated, and the resistance towards obesity and hepatic steatosis was observed. Involving the interaction between PPARγ and mammalian target of rapamycin (mTOR), ghrelin could activate its receptor on hepatocytes to promote lipogenesis as well [108]. Ghrelin also regulates insulin secretion and sensitivity in pancreatic β-cells and stimulates glucose output from primary hepatocytes [109]. Reported in a recent study [110], ghrelin reduced apoptosis induced by TNF-α and autophagy in human hepatocytes via AMPK/mTOR, consistent with another research where ghrelin showed anti-fibrosis effects by downregulating the TGF-β1/Smad3 signaling pathways and inhabiting autophagy [111].

Glucagon-like peptide-1 (GLP-1) is a hormone secreted by the enteroendocrine L-cells of the intestine, which causes potentiation of glucose-stimulated insulin secretion after nutrient intake. GLP-1 could downregulate the expressions of SREBP-1c, ACC, SCD-1, and FAS, which are all important players for de novo lipogenesis [112]. Hence, low level of GLP-1 in NAFLD populations possibly derived from producing dysfunction or cleavage enhancement is considered as one predisposing factor for NAFLD development [113]. Treatment of GLP-1 receptor agonists (GLP-1RA) significantly reduced blood glucose level, IR and hepatic lipid concentration in mouse steatohepatitis model [114]. GLP-1RA downregulated expression of SREBP-1c and SCD-1 and upregulated the expression of PPARα in hepatocytes, which could further suppress de novo lipogenesis and induce β-oxidation of free fatty acids [115]. The majority of clinical trials investigating the effects of GLP-1RAs on NAFLD patients has shown promising results [116, 117]. GLP-1RAs were able to significantly reduce hepatic steatosis as well as control blood glucose in NAFLD patients [118]. In a Chinese cohort, Liraglutide, a recombinant polypeptide analogue of GLP-1, was found superior to traditional therapies including metformin and gliclazide, in ameliorating hepatic steatosis, improving liver function, and controlling body weight [119]. Bile acids are released into the duodenum for lipid digestion and 95% of them are absorbed back into the portal circulation within the ileum for recycle. Increased bile acids in the intestine could activate the farnesoid X receptor (FXR) signaling in enterocytes and thereby induce FGF19 production and secretion. Besides their roles in regulating hepatic bile acid synthesis, FXR and FGF19 are also involved in metabolic homeostasis. Deletion of FXR led to significant increases in hepatic cholesterol and triglycerides [120, 121]. On contrary, the activation of FXR could prevent lipid accumulation in the liver by regulating hepatic de novo lipogenesis and fatty acid β-oxidation [122]. FGF19 inhibits the expression of lipogenic enzymes by increasing phosphorylation of signal transducer and activator of transcription 3 (STAT3) and decreasing the expression of PGC1β [123]. Peripheral administration of FGF19 could improve glucose tolerance in both HFD-feeding mice and ob/ob mice [124], possibly because of its motivating effects on hepatic protein and glycogen synthesis [125]. Although hepatic FXR protein contents and circulating FGF19 levels were found inversely associated with NASH severity in patients [126], more mechanistic studies are needed to clarify their roles in NAFLD development. As the agonist of FXR, obeticholic acid, has recently been studied in a multicenter, randomized, double-blind phase III study, and showed benefits in fibrosis improvement [127].

Gut harbors the most abundant bacterial populations in the body, while the importance of these ‘guests’ in human physiology has long been ignored. Recent studies have discovered more and more functional roles of gut microbiota, such as nutrient metabolism [129–131], xenobiotic metabolism [131, 132], antimicrobial protection [133], immunomodulation [134, 135] and gut integrity maintenance [136]. On this basis, the change in gut microbiota, or dysbiosis, is presumed associated with etiologies of many metabolic diseases, including NAFLD. Comparing individuals with NAFLD and non-NAFLD controls, both microbiome and bacterial density are different [137]. In NAFLD patients, increased intestinal permeability and bacterial overgrowth were reported positively correlated with the severity of steatosis [138, 139]. Germ-free mice were found resistant to DIO, in contrast to mice with the normal gut microbiota. Transplantation of stool from individuals with IR to healthy controls transfer the insulin resistant phenotype as well, which further highlights the contributions gut microbiota could make to host metabolic disorders [140].

Gut microbiota could ferment excess food that the host is not able to digest, and further produce short-chain fatty acids (SCFAs), including acetate, propionate, butyrate, etc., mostly in colon. Increased total SCFAs level is one common metabolic profile of both animal model and NAFLD patients, but precise production of SCFA is hard to measure so far in patients due to technical difficulties. One human study showed a significant association between the presence of steatohepatitis and an increased percentage Firmicutes and a reduced percentage of Bacteroidetes (two predominant bacterial phyla colonizing the healthy human large intestine) [141], and the increase of the Firmicutes/Bacteroidetes ratio was reported associated with increased energy harvest from the diet [142]. SCFAs not only provide extra energy to the host (about 30% of hepatic energy supply) [143] but also impact satiety and insulin signaling by stimulating the production of peptide YY (PYY) and GLP-1 in the intestine [144]. The insulin-mediated fat accumulation could be suppressed by the interaction of SCFAs and their receptors, G-protein-coupled receptors (GPCRs), in the gut, skeletal muscle, adipose tissue and the liver [145]. SCFAs stimulate the leptin production [146], increase adipogenesis and suppress lipolysis in adipose tissue [147]. Administration of SCFAs to mice and patients leads to increased energy expenditure [148, 149], thermogenesis [150] and fatty acids oxidation secondary to AMPK activation [151], but SCFAs also serve as substrates for lipogenesis and gluconeogenesis. There have been mixed reports regarding the roles of SCFAs in regulating inflammation. GPCRs could activate signaling pathways such as RAS, protein kinase A, phosphoinositide 3-kinases (PI3K), and extracellular regulated protein kinases (ERK1/2) and further upregulate the expressions of IL-1, IL-6, TNF-α, CXCL1, and CXCL2, in favor of the contribution of SCFAs in NASH pathogenesis [153–156]. Increased acetate level was reported responsible for production of inflammatory cytokines in macrophages and even inflammatory responses in the liver [156]. While under inflammatory conditions, administration of SCFAs reduces NF-κB activity via the inhibition of histone deacetylase (HDAC), which suppresses the production of inflammatory cytokines such as IL-6, IL-8, and TNF-α [155, 157, 158]. Furthermore, SCFAs could enhance differentiation of anti-inflammatory regulatory T cells (Tregs) in the colon, and also stimulate the NLRP3 inflammasome. In vitro, SCFAs also attenuate PPARγ activity and reduce the expression of lymphocyte function-associated antigen 3 (LFA3) and intercellular adhesion molecule 1 (ICAM1). The beneficial functions of SCFAs to prevent NAFLD was discussed by a variety of animal studies [160–163], but there still need to be direct evidences from clinical trials.

From saccharolytic fermentation, Proteobacteria (e.g., *Escherichia coli*) also produce a decent amount of ethanol and this ability could be increased by dysbiosis. In both obese mice and human subjects, higher breath ethanol contents were detected compared to controls, which could be abrogated by antibiotic treatment [163, 164]. In addition, elevated circulating ethanol levels and increased hepatic alcohol metabolism (in terms of alcohol dehydrogenase 1, aldehyde dehydrogenase 2 and Cytochrome P450 2E1) were also reported in NALFD patients [165]. Such increases of the endogenous ethanol not only negatively regulate gut environment, but also supply a constant source of ROS to the liver [166]. In HFD-fed mice, ethanol and free fat acids were showed to synergistically promote liver injury through the elevation of hepatic/serum free fatty acids and upregulation of the hepatic expression of several chemokines [167]. A recent work published in *Cell Metabolism* introduced a high-alcohol-producing *Klebsiella pneumoniae* (HiAlc *Kpn*) strain in a rare NASH case with bacterial auto-brewery syndrome and reported a strong correlation between HiAlc *Kpn* between NAFLD in a Chinese cohort. When a HiAlc-*Kpn*-strain-containing fecal microbiota was transplanted into normal mice, NAFLD also developed. These novel findings all together suggested the alteration in the gut microbiome could to some extent facilitate NAFLD development via excess production of endogenous ethanol [168].

Increased intestine permeability is another important alteration caused by gut dysbiosis. The relationship between gut permeability and NAFLD is highlighted by the finding in a high-fat dietary model of NAFLD that increased circulating Lipopolysaccharide (LPS) levels correlated with worsened steatohepatitis, as measured by the NAFLD Activity Score and liver enzyme levels [111]. Impaired gut barrier (unsealed junctions between intestinal endothelial cells) allows gut mucosal cells and the liver exposed to harmful substances derived from the gut, including translocated bacteria, LPS and endotoxins as well as secreted cytokines. Gut-derived bacterial products, especially LPS, could be recognized by Toll-like receptors (TLRs), which are important players in innate and adaptive immune responses [101]. Increased TLR ligands could be detected in the portal system in the presence of gut dysbiosis, which contributes to the activation of TLR4 on Kupfer cells and stellate cells and subsequent stimulation of pro-inflammatory and profibrotic pathways [13, 102, 169]. A range of signaling cascades, including MAPK, c-Jun N-terminal kinase (JNK), p38 mitogen-activated kinases and NF-κB, are involved in this process and lead to activation of proinflammatory genes, production of inflammasomes and release of ROS [13]. Activation of the NACHT, LRR and PYD domains-containing protein 3 (NLRP3) inflammasome by LPS from gut microbiota via TLR4 and TLR9 was reported necessary for NASH development since it led to the early onset of steatohepatitis. In liver samples from NASH patients, NLRP3 inflammasome was also found positively correlated with hepatic collagen type 1α expression [170]. Activation of TLRs could also contribute to hepatic steatosis via intestinal epithelial myeloid differentiation primary response gene 88 (MyD88) which is a central adaptor molecule for TLRS and is responsible for switching metabolism towards obesity [171].

### Brain and NAFLD

The central nerve system plays a predominating role in energy regulation as neuronal networks and nuclei in certain brain regions crosstalk and integrate peripheral signals like plasma nutrients and key metabolic hormones to coordinate adaptive changes in food intake and energy expenditure [172]. In particular, the hypothalamic arcuate nucleus (ARC) is considered as the most important central sensor for signals in circulation and cerebrospinal fluid because it is anatomically adjacent to the median eminence and the third ventricle [173]. Neurons in ARC are the first-order neurons on which peripheral metabolic hormones, including leptin, insulin, ghrelin and nutrients primarily act, and, they are each responsible for expressing orexigenic neuropeptides like neuropeptide Y (NPY) and agouti-related peptide (AgRP), or anorexigenic neuropeptides like proopiomelanocortin (POMC). Then, second-order neurons in the paraventricular nucleus (PVN), ventromedial hypothalamus (VMH) and lateral hypothalamus (LH) could receive signals from first-order neurons via axonal transport. AgPR released from NPY/AgRP neurons or α-melanocyte-stimulating hormone (α-MSH) released from POMC neurons could bind to the melanocortin-3 and -4 receptors (MC3R and MC4R) on second-order neurons. α-MSH activates catabolic pathways to modulate food intake and energy expenditure while AgPR competes with α-MSH for MC3Rs and MC4Rs and antagonizes its effects [174]. Deletion of MC4R in mice causes hyperphagia and obesity [175], and MC4R gene mutation is also associated with severe early-onset obesity in human study [176]. A novel mouse model composing both MC4R knockout and HFD feeding successfully simulates the clinical and pathologic features of human NASH. The loss of hypothalamic MC4R function accounts for the development of IR, dyslipidemia, liver fibrosis and HCC [177]. When ablating NPY/AgRP neurons in young mice, researchers find significant food intake inhibition and weight loss [178], and direct administration of NPY and AgRP also stimulate feeding in animal models [179]. PVN neurons synthesize catabolic neuropeptides and control autonomic outflow to peripheral metabolic organs, which increases fatty acid oxidation and lipolysis. Consistently, lesion study on PVN shows overeating and obesity in rats [180]. Neurons in the VMH could sense glucose and leptin and produce anorexigenic neuropeptides like brain-derived neurotrophic factor (BDNF). Destruction of VMH leads to hyperphagia, obesity and hyperglycemia [181]. In contrast, electronic or chemical ablation of the LH lead to anorexia and weight loss because LH neurons produce orexigenic neuropeptides melanin-concentrating hormone (MCH) and orexin which are responsible for interactions between ARC and LH. Chronic infusion of MCH causes obesity [182], and MCH deletion causes resistance to diet-induced obesity in mice [183, 184]. Recently, a selective MCH receptor 1 antagonist is reported successfully ameliorating obesity and hepatic steatosis in mouse NAFLD models [185].

By modulating energy consumption pathways (e.g., locomotor activity, fatty acid oxidation or thermogenesis), the brain also regulates energy expenditure of the body. The suprachiasmatic nucleus (SCN) produces tumor growth factor-α in a circadian manner to mobilize growth factor receptors in the hypothalamus paraventricular nucleus and subsequently inhibit [186]. LH neurons and POMC neurons also control locomotor activity by producing orexin and recognizing circulating signals like leptin level [173]. As mentioned above, thermogenesis is mainly carried out by BAT. The brain regulates BAT activity via the interaction of norepinephrine and β-adrenergic receptors. Downstream activation of cyclic-adenosine monophosphate (cAMP) signaling then upregulates mitochondrial uncoupling protein-1 (Ucp1), a key molecule for metabolic thermogenesis to avoid an excess of fat accumulation [187]. Hypothalamus integrates body temperature sensation and modulates thermogenesis with excitatory neurons in VMH and efferent sympathetic outflow. Abnormal hormonal signals could trick hypothalamus and influence sympathetic outflow to BAT [188]. Central administration of leptin, MC3/4R agonists, glucagon or GLP-1 could stimulate BAT activity. Intracerebroventricular co-injection of leptin and insulin induces WAT browning and then increases energy expenditure [189].

Particular of note, SCN is the master cerebral clock coordinating all biological clocks in the body and controlling [190]. The SCN pace the self‐sustained and cell‐autonomous molecular oscillators in the peripheral tissues through autonomic neural outputs and humoral signals such as melatonin and glucocorticoids [191]. A loss-of-function mutation in the *Clock* gene, a critical transcriptional factor affecting both the persistence and period of circadian rhythms, leads to hyperglycemia and dyslipidemia, and mice develop adipocyte hypertrophy and hepatic steatosis [192]. Under the circumstance of hepatic steatosis, clock-related gene alterations are found associated with pathways regarding fatty acid oxidation, lipoprotein, fatty acid synthesis and cholesterol metabolism. In the liver, immediate early genes are regulated by systematic signals and then communicate rhythmic signals to the hepatic molecular clockworks. The molecular clockwork provokes the expression of genes encoding the enzymatic and transport proteins managing lipogenesis and lipolysis, such as hepatic cytochrome P450 cholesterol 7α‐hydroxylase, 3‐hydroxy‐3‐methylglutaryl coenzyme A reductase (HMGCR), FAS, lipolytic enzymes, apoA‐IV and C‐III, low‐density lipoprotein receptor, FA transport protein 1 (FATP1), fatty acyl‐CoA synthetase 1 and adipocyte differentiation‐related protein [193].

Both the liver and the gut are rich in vagal afferent fibers which transmit local information to the brain stem, another key area involved in the central regulation of energy balance. In the brain stem, the nucleus tractus solitaries (NTS) is predominantly in charge of receiving afferent inputs indicating parameters of splanchnic organs and then processing extensive signals before delivering them to higher brain regions [194]. The NTS receives neuronal projections from the hypothalamus, the amygdala and the nucleus accumbens, and vice versa, hence, it serves as a relay station and presides over orchestrating a coherent output reflex to the periphery [195]. During HFD-induced obesity, the vagal afferent pathway becomes dysfunctional as the neuronal excitability is reduced. As a result, higher levels of stimulations (such as stomach distension or hormones) are needed to activate vagal afferents with reduced responding abilities [196]. Nevertheless, neurons of obese animal or human remain the normal phenotype as lean subjects irrespective of abundant energy stores, hence improper afferent signals could not be appropriately interpreted in the brain and will inevitably exacerbate hyperphagia and obesity by increasing meal consumptions and blunting satiety signals [197]. After phenol‐induced hepatic denervation in rats, CPT1 is suppressed. As a mitochondrial transport protein that traverses the outer mitochondrial membrane, CPT1 motivates fatty acids to enter the mitochondria, where the β-oxidation takes place [198]. In the hypothalamus, AMPK phosphorylates ACC, lowers malonyl‐CoA production, modulate CPT1 activity and thereby regulate energy balance. The AMPK-malonyl‐CoA-CPT1 axis has already been targeted for the therapy of NASH [199]. Interestingly, gut dysbiosis and relevant endotoxemia are considered partially contributing to the dysregulation of gut-brain vagal communication and subsequent outcomes such as obesity and NAFLD [200, 201]. In HFD-fed mice and obese patients, multiple hormones (CCK, PYY, GLP-1, etc.) become scarce capable to activate vagal afferents and their receptors in vagal neurons also reduce [196, 202, 203]. A posteriori, the benefits of bariatric procedures on NAFLD patients are also ascribed to the remediation of vagal nerve circuits [204].

Leptin activates receptors in many brain regions, among which ARC is the most important area to regulate appetite, thermogenesis, and locomotor activity [173]. Leptin drives hypothalamic signaling cascades such as the Janus kinase-STAT pathway, the insulin receptor substrate (IRS)-PI3K signaling, mTOR-S6 kinase signaling, AMPK signaling and ERK signaling [205]. Meanwhile, ghrelin also acts through central mechanisms to increase caloric intake and functions through hypothalamic neuronal circuits to increase lipogenesis and decreasing β-oxidation in WAT. By activating GHS-R1a on hypothalamic NPY/AgRP neurons, ghrelin promotes the blockade of MC3/4R and regulates peripheral lipogenesis through the sympathetic nervous system [206].

Hypothalamic neurons (such as orexin-producing neurons in the LH) could be activated by sweet foods [207], while one unique point to mention here is, besides the caloric and glycemic aspects, sweet taste itself should also be discussed when we talk about the role of brain in NAFLD and other metabolic disorders. When assigned to diet soda with aspartame, a nonnutritive sweetener worldwide consumed, rats develop hyperglycemia and fat accumulation in two months compared to their water drinking counterparts [208]. The aspartame exposure downregulates adiponectin and PPAR and increases leptin production, which may potentially contribute to the pathogenesis of NAFLD. A number of studies reported that consumption of artificial sweeteners brings great risks of obesity, metabolic syndrome and type 2 diabetes [209–212]. Reminded by these studies, the learned behavior of sweet taste is associated with disease pathogenesis. Oral but not gastric administration of glucose or saccharin leads to decreased GLP-1 release [213], which subsequently disrupts the satiety process and increases food intake and weight gain.

### Summary

**Fig. 1:**
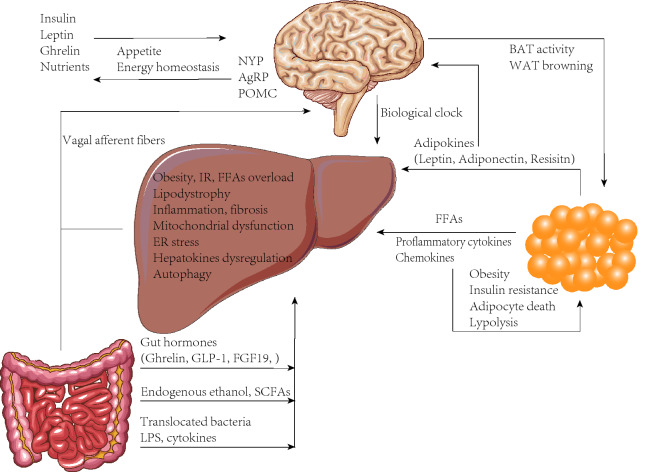
Inter-organ crosstalk regarding the pathogenesis mechanism of NAFLD. The adipose tissue contributes fatty acids to facilitate hepatic steatosis and also produces cytokines to impact proinflammatory pathways, which in turn exacerbates obesity, insulin resistance, adipocyte death and lipolysis. Adipokines deliver metabolic signals to the brain as well. Gut dysbiosis affects gut hormones, metabolites and bacterial components, which subsequently influence the development and progression of NAFLD. Moreover, gut hormones could also interact with different neurons of the brain, to influence appetite and energy homeostasis. The central nerve system integrates hormonal and neurol signals from peripheral organs to control energy balance, which when impaired leads to obesity and NAFLD. It also remarkedly influences lipogenesis and lipolysis by mastering biological clocks and modulating adipose activities. Consequently, in the liver, lipodystrophy may result in ROS production, mitochondrial dysfunction, ER stress, apoptosis, inflammation, hepatokine dysregulation, and autophagy, thus collectively inducing the development of NAFLD

NAFLD is a rising health problem worldwide and related medical burden is also increasing at an alarming rate. Recently, an international expert group has announced the consensus of renaming NAFLD as metabolic (dysfunction) associated fatty liver disease (MAFLD), in two position papers [214, 215]. Although there is still some feeling in academia that the new acronym is premature [216], the effort to bring this disease to a more practical status and closer to metabolic disorders is not doubt destined. The new terminology and corresponding diagnostic criteria explicitly highlight overweight/obesity, type 2 diabetes mellitus, and metabolic dysregulation, which shall bring more targets for mechanism research and intervention. Without doubt, the pathogenesis of NAFLD (or MAFLD) is complex and evidently involves multiple organs and diverse mechanisms (Fig. 1). The adipose tissue not only contributes fatty acids to facilitate hepatic steatosis but also produces hormones and cytokines to influence proinflammatory pathways. The gut directly takes charge of energy absorption and it communicates with brain to modulate food intake. Gut microbiota has become a hot topic recently as it participates in NAFLD pathogenesis via the gut-liver axis. Altered gut permeability also exposes liver to bacterial components and further triggers immune responses. The central nerve system integrates hormonal and neurol signals to control energy balance, which when impaired leads to obesity and NAFLD. Finally, in the liver, key mechanisms include lipotoxicity, IR, ROS production, ER stress, apoptosis, inflammation, autophagy, etc. Based on researches emphasizing aforementioned mechanisms, quests for novel therapies are underway and many candidates have already entered clinical trials. Further work is still urgently needed to find detailed pathogenic mechanisms of NAFLD, especially the inter-organ crosstalk aspect.

## Data Availability

Not applicable.

## Abbreviations

17-OHP: 17-Hydroxyprogesterone
4-HNE: 4-Hydroxynonenal
α-MSH: α-Melanocyte-stimulating hormone
ACC: Acetyl-CoA carboxylase
AG: Acylated ghrelin
AgRP: Agouti-related peptide
AMPK: AMP-activated protein kinase
ANGPTL8: Angiopoietin-like 8
ApoB: Apolipoprotein B
ARC: Arcuate nucleus
ASK1: Apoptosis signal‐regulating kinase 1
Atg7: Autophagy-related 7
BAT: Brown adipose tissue
BDNF: Brain-derived neurotrophic factor
cAMP: Cyclic-adenosine monophosphate
CCK: Cholecystokinin
CHOP: C/EBP homologous protein
CHIP: C-terminus Hsc70-interacting protein
ChREBP: Carbohydrate-responsive element-binding protein
CPT1: Carnitine palmitoyl transferase 1
DAG: Diacylglycerol
DGAT: Diglyceride acyltransferase
DIO: Diet-induced obesity
DNL: De novo lipogenesis
ECM: Extracellular matrix
EPI: Epinephrine
ER: Endoplasmic reticulum
ERK: Extracellular regulated protein kinases
FA: Fatty acid
FAS: Fatty acid synthase
FATP1: FA transport protein 1
FFAs: Free fatty acids
FGF19: Fibroblast growth factor 19
FGF21: Fibroblast growth factor 21
FOXO: Forkhead box O
FXR: Farnesoid X receptor
Gabarapl1: Gamma-aminobutyric acid type A receptor associated protein like 1
GHS-R: Growth hormone secretagogue receptor
GLP-1: Glucagon-like peptide 1
GLP-1RA: GLP-1 receptor agonist
GPCRs: G-protein-coupled receptors
GR: Glucocorticoid receptor
HCC: Hepatocellular carcinoma
HDAC: Histone deacetylase
HiAlc *Kpn*: High-alcohol-producing *Klebsiella pneumoniae*
HMGCR: 3‐Hydroxy‐3‐methylglutaryl coenzyme A reductase
HSCs: Hepatic stellate cells
ICAM1: Intercellular adhesion molecule 1
IKK: Inhibitor of κB kinase
IR: Insulin resistance
IRE1α: Inositol-requiring enzyme 1α
IRS: Insulin receptor substrate
JNK: Jun N-terminal kinase
LDs: Lipid droplets
LFA3: Lymphocyte function-associated antigen 3
LH: Lateral hypothalamus
LPS: Lipopolysaccharide
LXR: Liver X receptor
MAFLD: Metabolic (dysfunction) associated fatty liver disease
MAP3K: Mitogen‐activated protein kinase kinase kinase
MAPK: Mitogen-activated protein kinase
MC3R: Melanocortin-3 receptor
MC4R: Melanocortin-4 receptor
MCD: Methionine-choline deficient
MCH: Melanin-concentrating hormone
MDA: Malondialdehyde
MMP1: Matrix metalloproteinase 1
mTOR: Mammalian target of rapamycin
mtTFA: Mitochondrial transcription factor A
MTTP: Microsomal TAG transport protein
MyD88: Myeloid differentiation primary response gene 88
NAFLD: Non-alcoholic fatty liver disease
NASH: Non-alcoholic steatohepatitis
NE: Norepinephrine
NLRP3: NACHT, LRR and PYD domains-containing protein 3
NPY: Neuropeptide Y
NRF1: Nuclear respiratory factor 1
NTS: Nucleus tractus solitaries
PEPCK1: Phosphoenolpyruvate carboxy kinase 1
PGC1α: Peroxisomal proliferator-activated receptor gamma coactivator 1α
PI3K: Phosphoinositide 3 kinases
PKC: Protein kinase C
POMC: Proopiomelanocortin
PPAR: Proliferator-activated receptor
PRDM16: PR domain containing 16
PTMs: Posttranslational modifications
PVN: Paraventricular nucleus
PYY: Peptide YY
ROS: Reactive oxygen species
SCD1: Stearoyl-CoA desaturase 1
SCFAs: Short-chain fatty acids
SCN: Suprachiasmatic nucleus
Snap23: Synaptosomal associated protein 23
SOCS3: Suppressor of cytokine signaling 3
SREBP-1c: Sterol regulatory element-binding protein 1c
STAT3: Signal transducer and activator of transcription 3
T2DM: Type 2 diabetes mellitus
TAG: Triacylglycerol
TGF: Transforming growth factor
TIMP1: Tissue inhibitor of metalloproteinase 1
TLR: Toll-like receptor
TRAF2: Tumor necrosis factor receptor-associated factor 2
Tregs: Regulatory T cells
Ucp1: Uncoupling protein 1
UPR: Unfolded protein response
VLDL: Very low-density lipoprotein
VMH: Ventromedial hypothalamus
WAT: White adipose tissue
XBP1: X-box binding protein 1
